# Solar Radiation-Associated Adaptive SNP Genetic Differentiation in Wild Emmer Wheat, *Triticum dicoccoides*

**DOI:** 10.3389/fpls.2017.00258

**Published:** 2017-03-14

**Authors:** Jing Ren, Liang Chen, Xiaoli Jin, Miaomiao Zhang, Frank M. You, Jirui Wang, Vladimir Frenkel, Xuegui Yin, Eviatar Nevo, Dongfa Sun, Ming-Cheng Luo, Junhua Peng

**Affiliations:** ^1^Shandong Provincial Key Laboratory of Biophysics, Institute of Biophysics, Dezhou UniversityDezhou, China; ^2^Key Laboratory of Plant Germplasm Enhancement and Specialty Agriculture, Chinese Academy of SciencesWuhan, China; ^3^Department of Agronomy and the Key Laboratory of Crop Germplasm Resource of Zhejiang Province, Zhejiang UniversityHangzhou, China; ^4^Cereal Research Centre, Agriculture and Agri-Food CanadaWinnipeg, MB, Canada; ^5^Department of Plant Sciences, University of CaliforniaDavis, CA, USA; ^6^Department of Evolutionary and Environmental Biology, Institute of Evolution, University of HaifaHaifa, Israel; ^7^Department of Biotechnology, College of Agriculture, Guangdong Ocean UniversityZhanjiang, China; ^8^Department of Agronomy, College of Plant Science and Technology, Huazhong Agricultural UniversityWuhan, China; ^9^The State Key Lab of Crop Breeding Technology Innovation and Integration, China National Seed Group Co. Ltd.Wuhan, China

**Keywords:** genetic differentiation, solar radiation, natural selection, SNP marker, wild emmer wheat

## Abstract

Whole-genome scans with large number of genetic markers provide the opportunity to investigate local adaptation in natural populations and identify candidate genes under positive selection. In the present study, adaptation genetic differentiation associated with solar radiation was investigated using 695 polymorphic SNP markers in wild emmer wheat originated in a micro-site at Yehudiyya, Israel. The test involved two solar radiation niches: (1) sun, in-between trees; and (2) shade, under tree canopy, separated apart by a distance of 2–4 m. Analysis of molecular variance showed a small (0.53%) but significant portion of overall variation between the sun and shade micro-niches, indicating a non-ignorable genetic differentiation between sun and shade habitats. Fifty SNP markers showed a medium (0.05 ≤ *F*_*ST*_ ≤ 0.15) or high genetic differentiation (*F*_*ST*_ > 0.15). A total of 21 outlier loci under positive selection were identified by using four different *F*_*ST*_-outlier testing algorithms. The markers and genome locations under positive selection are consistent with the known patterns of selection. These results suggested that genetic differentiation between sun and shade habitats is substantial, radiation-associated, and therefore ecologically determined. Hence, the results of this study reflected effects of natural selection through solar radiation on EST-related SNP genetic diversity, resulting presumably in different adaptive complexes at a micro-scale divergence. The present work highlights the evolutionary theory and application significance of solar radiation-driven natural selection in wheat improvement.

## Introduction

Wild emmer wheat, *Triticum dicoccoides*, the progenitor of modern tetraploid and hexaploid cultivated wheats, is distributed over the Fertile Crescent and can be found in ecologically highly diverse environments (Peng et al., 2011; Chen et al., 2013; Ren et al., 2013b; Nevo, 2014). It consists of genomes AABB, resulting most probably from spontaneous hybridization of wild diploid einkorn wheat, *T. urartu* (2n = 2x = 14, genome AA), with a close relative of the goat grass *Aegilops speltoides* (2n = 2x = 14, genome SS, where S is closely related to B) (Dvorak and Zhang, 1990). Due to the ecological specialist and the only wild ancestor of cultivated wheat, wild emmer wheat is an excellent model organism for advancing evolutionary theory and wheat evolution (Nevo, 2014).

Recognition and understanding adaptive differentiation is an important and challenging task in evolutionary biology, due to that adaptation to the local environment is considered as a major driving force of phenotypic change and speciation (Volis et al., 2015). Local microscale natural laboratories are designated “Evolution Canyon” (EC) models by Nevo, and many studies on the “Evolution Canyon” model indicated that micro-geographic studies are excellent tests of local adaptive evolution and the evolutionary forces shaping evolution (Nevo, 2009, 2014, 2015; Venetsky et al., 2015; Huang et al., 2016). Ecological diversification between habitats favors local adaptation. Environmental factors, such as water availability, light intensity, soil composition as well as surrounding biota, may exert diverse selection that might drive evolutionary divergence of plant populations (Peleg et al., 2008; Ren et al., 2013a; Yin et al., 2015). Of all the environmental factors, solar radiation is perhaps the most spatially and temporally heterogeneous (Martínez-Ferri et al., 2001; Nevo, 2015). Solar radiation is associated with higher temperature and drought. The contrasted light environments can create different selective pressures; hence, habitat-based selection may have promoted divergence between populations with respect to genetic variability.

Adaptive genetic studies have been reported on both regional and local scales based on a dozen allozymes (Nevo et al., 1988), randomly amplified polymorphic DNAs (RAPD; Li et al., 1999), and microsatellites (SSRs; Li Y. et al., 2000; Li Y. C. et al., 2000; Li et al., 2002, 2003; Peleg et al., 2005; Volis et al., 2015; Yin et al., 2015) in wild emmer. All results indicated that allozymes, RAPDs, and SSRs were significantly correlated with ecogeographical stress variables, suggesting that both allozyme polymorphisms and DNA polymorphisms are at least partly adaptive and differentiated primarily by ecological factors such as alternative soils, topographies, or macro- and micro-climates in wild emmer wheat. However, studies focusing on adaptive genetic divergence and identifying genes or genomic regions involved in environmental adaptation, in particular using single nucleotide polymorphisms (SNPs), are less common in the wild emmer wheat. SNPs have become the most widely utilized molecular markers and are being extensively developed in crops, including wheat (Tiwari et al., 2014; MacCaferri et al., 2015; Voss-Fels et al., 2015; Shavrukov, 2016). SNP marker is now the most frequently used type of molecular marker for genetic variation in many species because of their high abundance across the genome and the availability of cost-effective high-throughput genotyping assays. More recent studies showed that SNP markers are appropriate for detecting selectively-channeled adaptive genetic differentiation in natural populations (Neafsey et al., 2008; Lamichhaney et al., 2012; Cavanagh et al., 2013; Ren et al., 2013a; Zhan et al., 2015; Cahill and Levinton, 2016). Besides, many literatures showed there are a lot of SNPs associated with effects of temperature or solar radiation in wheat (Beales et al., 2005; Eagles et al., 2011; Lei et al., 2013; Zhu et al., 2014).

Several approaches have been proposed to detect adaptive genetic variation by studying natural populations in contrasting environments without the need of trait phenotyping. Preferred methods to detect loci involved in local adaptation are based on the detection of “outlier” values of the allelic differentiation *F*_*ST*_. This *F*_*ST*_ approach has been proven to be an effective approach for studying adaptive genetic variation (Renaut et al., 2010; Prunier et al., 2011; Sim et al., 2011; Lamichhaney et al., 2012; Zhan et al., 2015). It was applied to many plants, such as tomato, boreal black spruce, and so on. The markers identified by using a *F*_*ST*_ -outlier method in these species tended to locate in genomic regions with known genes and quantitative trait loci (Prunier et al., 2011; Sim et al., 2011). However, utilization of *F*_*ST*_ approach to dissect wheat has not been reported so far.

In the present study, EST-related SNP markers were used to investigate adaptive genetic differentiation of wild emmer wheat from stressful abutting microclimatic niches at the Yehudiyya micro-site, Israel, and to analyze the relationship between ecological microclimates and DNA diversity. A *F*_*ST*_-outlier method was used to identify candidate loci associated with adaptation in the analyzed germplasm. Results of the present study will be useful in understanding the role of natural selection and distribution of genetic variation across the wheat genome.

## Materials and methods

### Plant materials

Germplasm of wild emmer wheat were collected in 1985 at the Yehudiyya located in an open oak forest of *Quercus ithaburensis* at the lower western foothills of the Golan Heights, northeast of the Sea of Galilee, Israel. The site is an area smaller than 1,000 m^2^ and included 12 repeated sampling plots (trees and their immediate circumference). Sampling was conducted in pairs in two solar radiation niches: (1) in shade, under the canopies of the oak trees (trees 10–20 m in height, with canopy diameters up to 20 m); (2) in sun, in the circumference around each tree and between trees. The sun-shade niches are abutting and the difference of the samples tested is 2–4 m apart. While (1) is largely shaded during the day, (2) is exposed in daytime to continuous sun radiation and drying. Hence, the soil temperature in the sun niche was almost 10°C higher than in the shade niche. During the growing season (October–May) of wild emmer wheat, the shade niche is under stresses of lower temperature and lower intensity of radiation in contrast to the sun niche. Besides, the two microniches vary significantly in their plant formation (Nevo et al., 1988). The shady microhabitat under the oak canopy harbors the plant formation of *Ricotia lunaria* and *Tordylium aegyptiacum*, with very sparse growth of other species (Nevo et al., 1988). The sunny microhabitat between the oak trees consists of the plant formation of *Psoralea hirsute, Carthamus glaucus, Ami majus, Olchoria pumilum, Eryngium creticum, Gundelia Tournefortii, Lavatera trimestris* with dense stands of wild cereals, *T. dicoccoides, Hordeum spontaneum*, and *Avena steritis*. Wild emmer is sparse in the shade under the oak canopy and abundant in the sun (Nevo et al., 1988). The microclimates vary significantly in the two microniches as reported previously (Nevo et al., 1988; Li et al., 1999, 2002). Therefore, the experimental design was done actually by nature. Each niche is the control of the other. In the present study, 92 individuals (47 from the shade and 45 from the sun) involving 12 trees were used for the advanced SNP genotyping.

### Genomic DNA extraction and SNP genotyping

Seeds of the genotypes used in this study were reproduced in 2001–2002. The genotypes were propagated twice and thus only two generations have passed since the first collection in 1985. Leaf samples of all genotypes were collected at seedling stage and frozen in liquid nitrogen. Genomic DNA was extracted from the young leaf samples using a modified SDS method as described in Ren et al. (2013b).

The 92 wild emmer wheat accessions were genotyped with 1,536 SNP markers. A detailed list of the 1,536 SNPs can be downloaded from the Wheat SNP Database (http://wheat.pw.usda.gov/SNP/new/index.shtml). Detailed information about SNP discovery, which was carried out on a panel of 32 lines of tetraploids and hexaploid wheat, has been described in a previous study (Akhunov et al., 2009). SNP selection and assay design were performed according to previously described procedures (Akhunov et al., 2009, 2010; Chao et al., 2010). Genotyping was performed at the UC Davis Genome Center (http://dnatech.genomecenter.ucdavis.edu/) using the Illumina Bead Array platform and Golden Gate Assay following the manufacturer's protocol.

SNP allele clustering and genotype calling was performed with the Illumina's Genome Studio (GS) V. 2010.3. In brief, the default clustering algorithm implemented in GS was first used to identify assays that produced three distinct clusters corresponding to the AA, AB, and BB genotypes expected for biallelic SNPs. Each SNP clusters was manually examined to correct imperfect calling of automated clustering. The accuracy for SNP clustering was validated visually (Akhunov et al., 2009; Chao et al., 2010).

### Statistical tests

In the present study, an estimate of genetic diversity was calculated for each locus, each chromosome and sub-population. The summary statistics including Nei's gene diversity and polymorphism information content (PIC) and heterozygosity were calculated by POWERMARKER Ver. 3.25 (Liu and Muse, 2005). Heterozygosity, defined as the identification of more than one allele for a given marker in a single accession, is simply the proportion of heterozygous individuals in the population (Liu and Muse, 2005). To test the significance of the differences in diversity among genomes and chromosomes, 95% confidence intervals (CI) of the genome mean for Nei's gene diversity and PIC were calculated, respectively, using bootstrap analysis with 1,000 replications. Chromosome means outside of the 95% CI were declared significantly different from the genome mean (Ren et al., 2013b).

The population differentiation was assessed with an analysis of molecular variance (AMOVA) using the ARLEQUIN version 3.11 software with 16,000 permutations (Excoffier and Lischer, 2010).

Forward stepwise discriminant analysis was performed to distinguish individuals from the two microclimatic niches according to the allele's frequencies, also implemented in the SPSS ver. 13.0 software (http://www.spss.com).

Narum and Hess (2011) suggested that combining results of multiple algorithms should be employed to minimize the type I (false positive) and type II (false negative) errors when using *F*_*ST*_ outlier test for detection of regions/markers under selection. Therefore, we used the following four different algorithms to detect selection footprints. The first *F*_*ST*_ outlier test approach implemented in LOSITAN was used to identify outlier loci (Antao et al., 2008). Outlier values of *F*_*ST*_ were identified from a plot of *F*_*ST*_ vs. heterozygosity that was generated under a simple island model. The analysis was performed with 100,000 simulations using an infinite allele model. We used a strict threshold of 0.99 and a false discovery rate of 0.05 to minimize the number of false positives.

Secondly, ARLEQUIN software was used to detect outlier loci that are putatively under selection (Excoffier and Lischer, 2010). In the program, the hierarchical structure among populations revealed by the results of the clustering was taken into account to avoid possible false positives. Each population was assigned to a group based on the average proportion of membership (Q) calculated from STRUCTURE analysis with neutral markers (Pritchard et al., 2000). Populations were assumed as admixed if *Q* < 0.7. These populations with *Q* < 0.7 were removed before outlier test by ARLEQUIN. We ran 20,000 simulations assuming 100 demes per group in this analysis. The joint null distribution of *F*_*ST*_ and heterozygosity [heterozygosity within populations divided by (1- *F*_*ST*_)] was obtained according to Excoffier and Lischer (2010). The significance level chosen was 0.01, hence, candidate loci under positive selection were selected based on *F*_*ST*_ values that fall outside of the 99% confidence interval.

The third algorithm for detecting outlier SNPs was implemented in BayeScan 2.1 (http://www.cmpg.unibe.ch/software/bayescan/) using the Bayesian likelihood method via reversible-jump Monte Carlo Markov chain (MCMC). This method is based on a logistic regression model that separates locus-specific effects of selection from population-specific effects of demography. BAYESCAN runs were implemented using default values for all parameters, including a total of 100,000 iterations after an initial burn-in of 50,000 steps. A threshold for posterior odds (PO) > 10 (strong selection) was used as a marker to be considered under selection. This corresponds to a posterior probability >0.91 for the model accounting for selection.

The fourth algorithm for detecting outlier SNPs was permutation test (https://en.wikipedia.org/wiki/Resampling_(statistics)#Permutation_tests). The method is described as the following. The *F*_*ST*_ value was calculated for each marker in given subdivision of plants in sunny and shady niches using observed allele frequencies and numbers of plants with non-missed genotype (e.g., https://en.wikipedia.org/wiki/Fixation_index). *F*_*ST*_ value for the given subdivision of plants was compared with ones obtained for plants randomly subdivided into two groups. To take this multiple comparison problem into account, FDR correction for *p*-values of individual markers was applied (Benjamini and Hochberg, 1995). The number of permutation run and a false discovery rate was set as 10,000 and 0.005, respectively.

## Results

### SNP genotyping and genomic distribution

Genotyping of 92 wild emmer accessions from Yehudiyya with multiplexed 1,536 Illumina Golden Gate SNP assay generated 141,312 genotypic data points. After removal of the SNPs failing to generate clear genotype clustering, 1,360 SNPs with high-quality genotype calls were detected. Six hundred and ninety-five of the 1,360 successful assays were monomorphic across all the 92 accessions and the overall polymorphism rate was 51%. The 695 polymorphic SNP markers were used for further data analyses. Table 1 shows the marker distribution, Nei's gene diversity, PIC values, and heterozygosity calculated for each chromosome and genome.

**Table 1:** **Genomic distribution and diversity index of 695 polymorphic SNP markers in wild emmer wheat population at Yehudiyya**.

**Chromosome**	**No. of polymorphic markers**		**Gene diversity**		**PIC**		**Heterozygosity**	
	**A genome**	**B genome**	**A genome**	**B genome**	**A genome**	**B genome**	**A genome**	**B genome**
1	58	57	0.1756	0.1934	0.1463	0.1631	0.1649	0.1558
2	53	45	0.1620^*^	0.1913	0.1400^*^	0.1617	0.0984^*^	0.1558
3	49	39	0.1888	0.2046	0.1600	0.1683	0.1453	0.1661
4	65	36	0.1857	0.1620^*^	0.1547	0.1386^*^	0.1856^*^	0.1286^*^
5	36	29	0.1986	0.1975	0.1668	0.1671	0.1473	0.1402
6	60	65	0.1989	0.2148^*^	0.1679^*^	0.1805^*^	0.1361	0.1007^*^
7	54	53	0.1758	0.1731	0.1494	0.1468^*^	0.1465	0.1009^*^
Total/Mean	373	322	0.1836	0.1922	0.1549	0.1619	0.1479	0.1334

* *outside of the 95% bootstrap confidence interval of the genome mean*.

Polymorphic SNP loci were not evenly distributed across the 14 chromosomes. The coverage, number of marker loci per chromosome, ranged from 29 in chromosome 5B to 65 in chromosome 4A and 6B (Table 1). Nei' gene diversity ranged from 0.1620 in chromosome 2A and 4B to 0.2148 in chromosome 6B with an average of 0.1876. The PIC values varied from 0.1386 in chromosome 4B to 0.1805 in chromosome 6B with an average of 0.1619. Differences among chromosomes were significant (*P* < 0.05) for gene diversity and PIC (Table 1).

Of the polymorphic loci, 373 were located on the A genome and 322 on the B genome. However, greater genetic variation was found in genome B than that in genome A (Table 1). Nei's gene diversity and PIC values for the A genome were 0.1836 and 0.1549, and those for the B genome were 0.1922 and 0.1619, respectively. Although, all the A chromosomes were mapped with more markers than the corresponding B chromosomes, for most (4/7) of the seven homoeologous groups, the genetic diversity in B chromosomes is obviously higher than that for the corresponding A chromosomes (Table 1).

As shown in Table 1, heterozygosity was higher in A genome (0.1479) than the B genome (0.1334). In A genome, heterozygosity varied from 0.0984 in 2A to 0.1856 in 4A chromosome. In B genome, heterozygosity varied from 0.1007 in 6B to 0.1661 in 3B chromosome. Thus, heterozygosity varied among the genomes and the chromosomes (Table 1).

### Genetic diversity between shady and sunny niches

Relative to the shady microclimatic niches, a higher level of genetic variation was detected in the sunny microclimatic niches. Based on 695 polymorphic markers, average Nei's gene diversity and PIC were 0.1879 and 0.1577 in the sunny niches, and 0.1843 and 0.1551 in the shady niches, respectively (Table 2). For most of the homoeologous groups and the two genomes, the genetic diversity in the sunny niche is obviously higher than that in the shady niches. However, the average of heterozygosity was slightly higher in shady niches (0.1435) than that in the sunny niches (0.1388), indicating a better outcrossing conditions in the shady niche (Table 2).

**Table 2:** **Genomic distribution and comparison of genetic diversity generated by 695 polymorphic SNP markers in wild emmer wheat population between shady and sunny niches at Yehudiyya**.

	**No. of SNP markers**	**No. of polymorphic markers**		**Gene diversity**		**PIC**		**Heterozygosity**	
		**Shady**	**Sunny**	**Shady**	**Sunny**	**Shady**	**Sunny**	**Shady**	**Sunny**
**CHROMOSOME**									
1	212	86 (40.6%)	110 (51.9%)	0.1786	0.1883	0.1486	0.1579	0.1599	0.1610
2	181	87 (48.1%)	90 (49.7%)	0.1713	0.1757	0.1457	0.1496	0.1285	0.1209
3	165	73 (44.2%)	84 (50.9%)	0.1915	0.1974	0.1603	0.1639	0.1591	0.1497
4	199	83 (41.7%)	96 (48.2%)	0.1702	0.1832	0.1427	0.1532	0.1683	0.1622
5	159	61 (38.4%)	61 (38.4%)	0.1962	0.1967	0.1657	0.1641	0.1493	0.1388
6	230	105 (45.7%)	120 (52.2%)	0.2089	0.2002	0.1758	0.1681	0.1198	0.1154
7	236	88 (37.3%)	102 (43.2%)	0.1728	0.1742	0.1467	0.1467	0.1235	0.1244
**GENOME**									
A	765	315 (41.2%)	351 (45.9%)	0.1810	0.1832	0.1527	0.1536	0.1502	0.1454
B	595	265 (44.5%)	308 (51.8%)	0.1881	0.1933	0.1578	0.1624	0.1357	0.1311
Total	1360	580 (42.6%)	659 (48.5%)						
Grand mean				0.1843	0.1879	0.1551	0.1577	0.1435	0.1388

**Figure 1:**
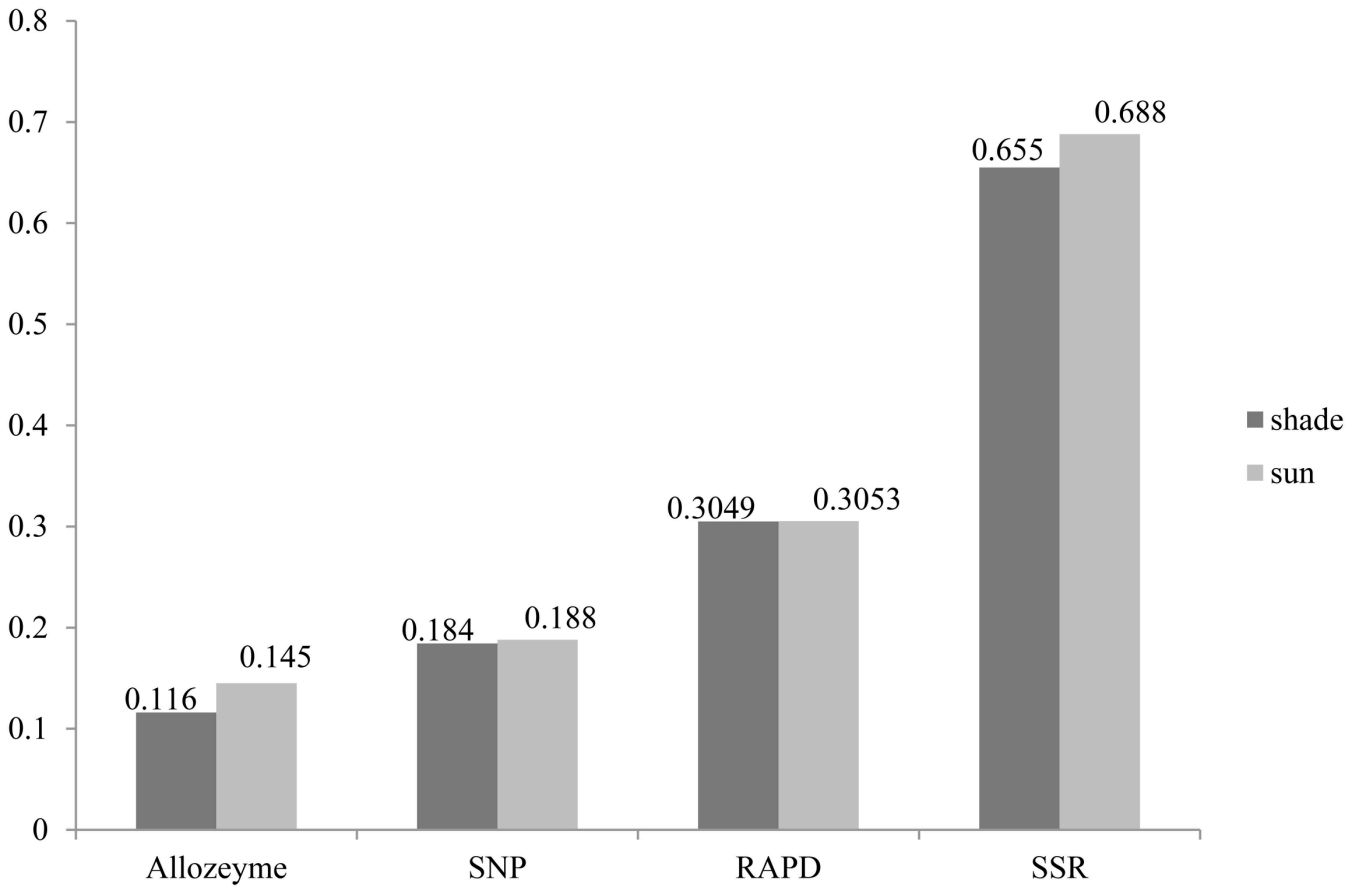
**Gene diversity profiles of allozyme, RAPD, SSR, and EST—related SNP loci in wild emmer wheat population at Yehudiyya**.

Similarly, the higher polymorphic level obtained from the sunny niches also reflect greater genetic variation in comparison with that in the shady niches. Of the 1,360 SNPs markers, 580 (42.6%) and 659 (48.5%) polymorphic markers were detected in shady and sunny niches, respectively. And also for all the seven homoeologous groups and the two genomes, the polymorphism level in the sunny niche is obviously higher than that in the shady niches (Table 2). This result demonstrated that a panel of shady niches has a relatively lower level of genetic diversity than the panel of sunny niches. These results, revealed by SNP markers, are obviously consistent with that of previous studies using the allozymic, RAPD, and SSR markers (Figure 1; Nevo et al., 1988; Li et al., 1999, 2002). These consistent genetic differences are from sample pairs of only a few meters apart (2–4 m).

### Divergence revealed by EST-related SNP markers between shady and sunny niches

A total of 695 markers were found to be polymorphic in the panel of 92 wild emmer accessions. Among these SNP markers, 115 were polymorphic only in the sun, while 36 were polymorphic only in the shade, and the remaining 544 markers were polymorphic in both shade and sun. The locus-by-locus *F*_*ST*_ estimates for the 695 SNP markers ranged from −0.011 to 0.223 between the shady and sunny microclimatic niches and showed a global estimate of 0.0053 (Table 3). Although, the global estimate indicated that small genetic differentiation was attributed to differences between the two micro-niches, the exact test revealed a significant micro-environment differentiation (*P* = 0.00842; Table 3). Furthermore, a total of 50 SNP markers showed a medium (0.05 ≤ *F*_*ST*_ ≤ 0.15) or high genetic differentiation (*F*_*ST*_ > 0.15).

**Table 3:** **Analysis of molecular variance (AMOVA) between shady and sunny niches at Yehudiyya**.

**Source of variation**	**Sum of squares**	**Percentage of variation (%)**
Among populations	97.354	0.529
Within populations	11896.587	99.471
Total	11993.940	100
Fixation Indices, *F*_*ST*_ = 0.00529, *P =* 0.00842		

*P-value was obtained through significance test using 16,000 permutations*.

Forward stepwise discriminant analysis was performed to distinguish individuals from the two microclimatic niches according to 50 divergent markers between the shady and sunny micro-niches. The result indicated that six (BQ171182_6_B_Y_188, BQ167580_3_A_Y_342, BE404341_5_B_Y_124, BE590553_7_B_Y_165, BE489244_3_A_392, BE591273_7_A_Y_335) of the 50 SNP markers were sufficient to correctly classify 81.5% of 92 individuals into their original microclimatic sunny or shady niches, whereas only ten (21.3%) plants from the shade were incorrectly assigned to the sun, and seven (15.6%) individuals from the sun were incorrectly assigned to the shade. The difference between the distribution center of the two subpopulations was highly significant [*F*_(6, 85)_ = 11.246, *P* < 0.0001]. Even using three (BE489244_3_A_392, BQ171182_6_B_Y_188, BE590553_7_B_Y_165) of the 50 SNP markers, 72.8% of 92 individuals could be correctly assigned to their original micro-niches, and only 13 plants (27.7%) from the shade and 12 plants (26.7%) from the sun were incorrectly classified into the alternative niche. Here too, the difference between the centroids of the two micro-niches was also highly significant [*F*_(3, 88)_ = 11.129, *P* < 0.0001]. These results clearly suggested obvious genetic differentiation at some loci between the two microclimatic niches and that the observed genetic differentiation at SNP loci is substantial, niche-associated, and therefore, ecologically determined.

### Candidate loci under positive selection

**Figure 2:**
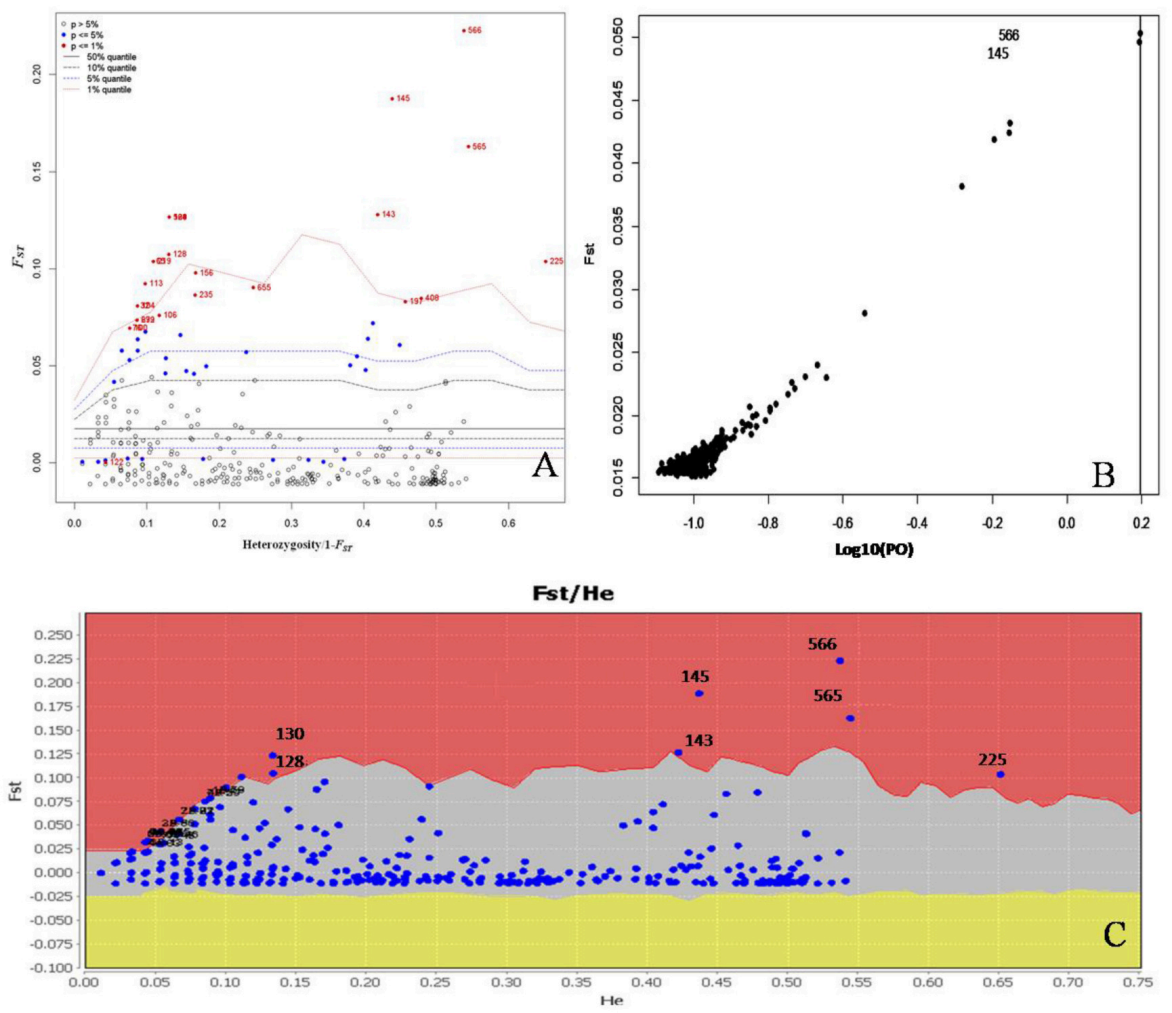
**Detection of outlier SNPs under positive selection using multiple algorithms. (A)** ARLEQUIN 3.5., *F*_*ST*_, locus-specific genetic divergence among populations; Heterozygosity/1- *F*_*ST*_, a modified measure of heterozygosity per locus. Loci significant at the 1% level are indicated by red dots. (B) BayeScan. *F*_*ST*_ is plotted against the log10 of the posterior odds (PO). The vertical line shows the critical PO used for identifying outlier markers. The two markers on the right side of the vertical line are candidate loci under positive selection. **(C)** LOSITAN. loci under selection above the 99% percentile (red area), neutral markers (lighter shaded area) and markers under balancing selection (yellow area).

We conducted further analyses to identify candidate loci that are under positive selection between the two microclimatic niches. As Narum and Hess (2011) recommended, four different *F*_*ST*_ outlier detection algorithms were used, i.e., ARLEQUIN, LOSITAN, BayeScan, and permutation test, in this study. In ARLEQUIN, a total of 18 outlier loci were revealed as the candidates subjected to positive selection (Table 4; Figure 2A). LOSITAN identified 19 outliers putatively under directional selection with a significance level of 0.99 (Table 4; Figure 2C). Between these two algorithms, 16 outlier SNP loci were repeatedly detected and five were detected by either of them. Thus, a total of 21 outlier SNP loci were identified by using ARLEQUIN and LOSITAN, and majority (76% = 16/21) were reproducible between these two algorithms (Table 4; Figures 2A,C).

**Table 4:** **ESTs and plausible functions in homologous ESTs outlier loci under positive selection between shady and sunny niches at Yehudiyya**.

	**SNP marker and the EST**			**Gene function and homologous EST**			
**Code**	**Marker**	**Accession No**.	**Map position (Bin)**	**Function**	**Accession No**.	**Identity (%)**	**E value**
Outlier 1⋆♦	BE443588_1_A_65	BE443588	1A	DNA methyltransferase 1-associated protein 1-like	XM_003567945.1	91	0
Outlier 2⋆♦	BG606586_1_B_Y_134	BG606586	C-1BL6-0.32	Aldose reductase-related protein	X57526.1	93	0
Outlier 3⋆	BE444305_1_B_433	BE444305	1BL2-0.69-0.85	Protein IQ-DOMAIN 31-like	XM_003567968.1	83	5e-120
Outlier 4⋆♦	BE590634_1_B_338	BE590634	1BL1-0.47-1.00^*^	Farnesyl pyrophosphate synthase-like	XM_003567928.1	91	0
Outlier 5⋆♦	BE405604_2_A_Y_353	BE405604	C-2AL1-0.85	NudC domain-containing protein 2-like	XM_003579541.1	91	9e-52
Outlier 6⋆♦	BF201533_2_A_Y_120	BF201533	2A	Unknown			
Outlier 7⋆♦•	BE426620_2_A_Y_420	BE426620	2AL1-0.85-1.00	Glutamyl-tRNA(Gln) amidotransferase subunit A-like	XM_003580613.1	93	0
Outlier 8^*^⋆▴♦•	BE585760_2_A_Y_481	BE585760	C-2AL1-0.85	PAL(phenylalanine ammonia-lyase) gene	X99725.1	93	0
Outlier 9⋆♦	BE498892_2_A_208	BE498892	2AL1-0.85-1.00	Pyruvate kinase, cytosolic isozyme-like	XM_003580774.1	91	0
Outlier 10⋆♦	BE499251_2_A_N_239	BE499251	2AL1-0.85-1.00	Elicitor-responsive protein 3-like	XM_003579445.1	83	3e-97
Outlier 11⋆	BE489244_3_A_392	BE489244	3AS4-0.45-1.00	VHS domain-containing protein At3g16270-like	XM_003565981.1	92	0
Outlier 12⋆♦	BG263601_4_A_N_448	BG263601	4AL4-0.80-1.00	Aldo-keto reductase/ oxidoreductase	EU971604.1	85	5e-129
Outlier 13^*^⋆♦	BG604507_4_B_383	BG604507	4B	Chloroplast fructose-bisphosphate aldolase(FBA)	FJ625793.2	98	0
Outlier 14⋆♦	BE590521_6_B_N_331	BE590521	C-6BL3-0.36	Adenine phosphoribosyltransferase 2-like	XM_003575301.1	90	4e-75
Outlier 15⋆♦•	BE426413_6_B_286	BE426413	C-6BL5-0.40^*^	Adenosine kinase 2-like	XM_003575347.1	94	0
Outlier 16⋆▴♦•	BQ171182_6_B_Y_188	BQ171182	6BL5-0.40-1.00	Unknown protein			
Outlier 17⋆♦	BE493897_7_B_Y_94	BE493897	7BS1-0.27-1.00	Bifunctional dihydroflavonol 4-reductase/flavanone 4-reductase-like	XM_003572334.1	90	0
Outlier 18⋆♦	BF293181_7_B_54	BF293181	7B	Dihydroflavonol-4-reductase-like, transcript variant 1	XM_003563553.1	88	7e-178
Outlier 19♦	CD453617_6_B_Y_138	CD453617	6BL5-0.40-1.00	AP-1 complex subunit gamma-2-like	XM_003570728.1	89	2.00E-179
Outlier 20♦	BE471213_6_A_N_28	BE471213	6AL8-0.90-1.00	Metal tolerance protein C2-like	XM_003570688.1	92	6.00E-178
Outlier 21♦	CD453605_6_B_427	CD453605	6B	Putative nitric oxide synthase-like	XM_003570728.1	89	2.00E-179

* *Candidate loci from known genes in wheat; ⋆▴, ♦, and • represent outliers identified using ARLEQUI, BayeScan, LOSITAN, and permutation test, respectively*.

**Figure 3:**
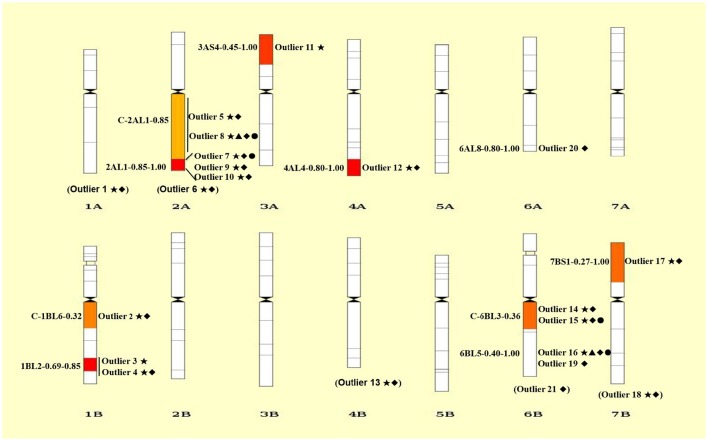
**Chromosomal distributions of 21 outlier loci under positive selection**. The intervals are shown on the left and the codes of mapped loci are indicated on the right of each chromosome. Details of codes are showed in Table 4. The outliers without knowing the exact bin in the chromosome are showed in parentheses at the bottom of each chromosome. Only the bins with mapped SNP loci are shown. ⋆, ▴, ♦, and • represent outliers identified using ARLEQUI, BayeScan, LOSITAN and permutation test, respectively.

To enrich our analysis, we used other two different algorithms, BAYESCAN and permutation test, to further check the outlier loci. A small number of outlier loci were detected when using BAYESCAN (two outliers, outlier 8 and outlier 16) and permutation test (four outliers: outlier 7, outlier 8, outlier 15, and outlier 16; Table 4). The two outliers detected by BayeScan (Figure 2B) were among the four identified by the permutation test. These four outliers were included in the 16 outliers reproducible between the ARLEQUIN and LOSITAN (Table 4; Figure 3). Obviously, BAYESCAN and permutation test are very reliable but also very conservative algorithms in detection of *F*_*ST*_ outlier loci under selection.

Collectively, a total of 21 outlier loci putatively under selection were identified. Genomic locations of these loci were presented in wheat chromosome bin maps (Figure 3). Among the 21 loci under positive selection, most of which (14/21 = 66.67%) were mapped in chromosomes 1B, 2A, and 6B (Table 4; Figure 3).

Since the presented SNP markers were discovered directly from the EST sequences or from genomic sequences amplified using PCR primers designed from ESTs, a putative function may be assigned to the underlying genes based on a comparison to a protein sequence database. Corresponding EST information of the 21 candidates and homology information of the ESTs were shown in Table 4. Six of the 21 proteins including aldose reductase-related protein, phenylalanine ammonia-lyase (PAL), elicitor-responsive protein, aldo-keto reductase/oxidoreductase, and chloroplast fructose-bisphosphate aldolase (FBA), which play important roles in plant responses to biotic and abiotic stresses in wheat, were identified as under positive selection among microclimatic niches. Especially, enzyme activity of FBA detected in wheat was obviously influenced by temperature and light (Lv et al., 2011; Cai et al., 2016).

## Discussion

### Genetic variation revealed by EST-related SNP markers

In this study, average Nei's gene diversity for shady and sunny microclimatic niches were 0.1843, and 0.1879, respectively (Table 2). Compared with the previous studies using SSR (shade: 0.655, sun: 0.688; Li et al., 2002), RAPD (shade: 0.3049, sun: 0.3053; Li et al., 1999), and allozyme (shade: 0.116, sun: 0.145; Nevo et al., 1988), the level of genetic diversity revealed by SNP markers is not very high (Figure 1). This can be expected due to the more conserved nature of the coding sequences sampled by EST-related SNP markers relative to non-coding sequences sampled by SSRs and RAPDs. Another explanation may be the property of SNP and the definition of gene diversity. SNPs are bi-allelic markers and, therefore, are limited to maximum PIC values of 0.5, whereas multi-allelic markers (e.g., SSR) do not have this limitation.

Despite the above-mentioned nature of SNP markers, it has been confirmed that EST-related SNP markers have adequate levels of polymorphisms for genetic evaluation in wheat. All results revealed by allozyme, RAPD, SSR, and SNP markers demonstrated a good agreement on the relative level of genetic diversity, i.e., higher in the sun than in the shade (Figure 1). Also, the greater genetic variation was detected in the B genome relative to the A genome, suggesting that the B genome has larger contribution to genetic variation than the A genome (Table 1). The different contribution of A and B genome to genetic variation agrees well with previous studies by using SSR markers (Röder et al., 1998), RFLP markers (Liu and Tsunewaki, 1991), and AFLP markers (Peng et al., 2000) within common hexaploid wheat as well as in wild emmer wheat (Peng et al., 2011; Ren et al., 2013a). These results suggested a sufficient level of variation when using SNP markers to carry out analyses of genetic variations and association mapping in wild emmer wheat. Hence, it is anticipated that SNP markers will play an increasingly important role in research of wheat genetics and breeding applications.

### Differentiation and adaptation within and among microclimatic niches

AMOVA based on 695 polymorphic SNP markers showed that genetic variation within microclimatic niches of wild emmer wheat is obviously larger than that among microclimatic niches, 99.47 vs. 0.53% (Table 3). However, the genetic variance between the two microclimatic niches or fixation index (*F*_*ST*_ = 0.053), was highly significant (*P* = 0.00831) as indicated by permutation test (Table 3). Furthermore, significant population differentiation was detected at 50 loci between the shady and sunny micro-niches, with *F*_*ST*_ > 0.05. Therefore, the differentiation between the two microclimatic niches, sun and shade, has truly occurred at a distance of a few meters apart (2–4 m), suggesting divergent adaptive complexes in sun and shade.

It is known that gene flow is a conservative homogenizing factor that prevents the divergence of only partially isolated populations (Nevo, 2011). If there was no strong microclimatic selection in such a small area of only a few meters between the sunny and the shady niches, a small amount of migration or gene flow would be sufficient to swamp the differentiation. The observed SNP differentiation in the present study might not have been maintained, as suggested by Li et al. (1999, 2002) who showed how selection overrules gene flow, as is also the case at “Evolution Canyon” (Nevo, 2011, 2014).

Natural selection drives local adaptation, potentially even at small temporal and spatial scales. As a result, adaptive genetic divergence can occur among populations living in different habitats. In the present study, the soil temperature in the sunny niche was almost 10°C higher than in the shady niche, as shown in Nevo et al. (1988) and Li et al. (1999, 2002). Since microclimates vary significantly in the two abutting micro-niches, such local microclimate differentiation across a few meters may enhance plant populations to evolve local ecological adaptations that provide an advantage under the prevailing conditions (Nevo, 2001; Peleg et al., 2008). Furthermore, the specific adaptations of the soil fungus *Aspergillus niger* to different solar radiation were investigated and the results indicated that mean conidial melanin concentration of strains from sunny microniches were threefold higher than strains from shady microniches, suggesting that melanin in *A. niger* is an adaptive trait against solar ultraviolet radiation generated by natural selection (Singaravelan et al., 2008). Therefore, genetic differentiation between the microclimatic niches may be mainly due to microclimatic diversifying natural selection. According to Li et al. (1999, 2002) and Nevo (2011, 2014), we assume that the revealed patterns may be regarded as an adaptive strategy for increasing fitness in their alternative ecologies.

SNP differentiation observed in the present study reflects an adaptation to microclimatic specificity of the two very close microclimatic niches, as previously shown by allozymic polymorphism (Nevo et al., 1988), RAPD (Li et al., 1999), and SSR (Li et al., 2002). The entire genome, both coding and noncoding regions, are subjected to the microclimatic natural selection.

### Candidate SNP loci subjected to positive selection

Although, a variety of molecular markers, mainly allozyme, RFLP and SSR, were already used to investigate microclimatic stress and adaptive DNA differentiation between the abutting shady and sunny niches in wild emmer wheat at Yehudiyya (Nevo et al., 1988; Li et al., 1999, 2002), EST-related SNP markers can provide additional perspectives. EST-related SNPs were derived from genomic sequences amplified from conserved primers, which were located in exons and were designed on the conserved sequences between wheat EST and rice genomic sequences (You et al., 2009; Akhunov et al., 2010). The polymorphisms can be used to directly map functional, expressed genes, rather than DNA sequences derived from conventional RAPD and AFLP marker techniques, which are typically not genes (Table 4; Figure 3). Thus, the SNP markers provides the opportunity to investigate local adaptation in natural populations and identify candidate genes under positive selection.

The identification of loci that have undergone positive selection is a fundamental step in understanding local adaptation. In the present study, four different *F*_*ST*_-testing algorithms were used to detect candidate SNP markers under selection of solar radiation-associated pressure. There are 18 and 19 outliers detected using ARLEQUIN and LOSITAN, respectively, and 16 of which are common between the two algorithms (Table 4; Figure 3). Based on permutation test, we identified four outliers (outlier 7, outlier 8, outlier 15, and outlier 16), belonging to a subset of the 16 common outliers detected by both ARLEQUIN and LOSITAN. BayeScan generally identified fewer outliers, only outlier 8 and outlier 16 in this study. These two BayeScan outliers are also detected by all other three algorithms, ARLEQUIN, LOSITAN, and permutation test, and thus are extremely reliable. Overall, we obtained consistent results using four different algorithms in our *F*_*ST*_ outlier test, and variation in the number of outliers among the algorithms (Table 4; Figure 3). The main reason for the number difference could be that these algorithms are based on completely different underlying models and mathematical approaches (Narum and Hess, 2011; Mita et al., 2013; Lotterhos and Whitlock, 2014; Feng et al., 2015). *F*_*ST*_ within-populations is variable in BayeScan (Foll and Gaggiotti, 2008), but is assumed to be the same across all populations in the LOSITAN program (Antao et al., 2008). The hierarchical structure among populations revealed by the results of the clustering is taken into account in the ARLEQUIN (Excoffier and Lischer, 2010). The combined inference from these algorithms that are based on completely different underlying models and mathematical approaches provides robust statistical support for the divergent pattern of differentiation at some loci between the two microclimatic niches, separated by only a few meters of distance.

It is worthy of pointing out that the candidate loci were mainly clustered in a few of chromosomal regions, suggesting that there are “hot spots” for directional selection in wild emmer wheat population at the Yehudiyya site. For instance, outlier 3 and 4, both were localized in chromosome 1BL2-0.69-0.85; similarly, outlier 7, 9, and 10 were mapped together into the 2AL1-0.85-1.00 (Table 4; Figure 3). Furthermore, the identified candidate loci have a disproportional bias with 66.7% mapped to chromosomes 2AL, 1BL, and 6BL (Table 4; Figure 3). It must be noted that association analysis of agronomic traits with SNP markers in durum wheat in our previous study has showed that several associations also locate in the same chromosome regions, and association clusters were identified on chromosome arms 2AL, 1BL, and 6BL (Hu et al., 2015). Similarly, some obvious QTL clusters for domestication traits were mapped to chromosome arms 2AL, 1BL, and 6BL by Peng et al. (2003) in wild emmer wheat. More studies have shown that genes for resistance often reside in clusters in wheat genome, and chromosome intervals 1BL2-0.69-0.85, 2AL1-0.85-1.00, and C-6BL3-0.6 are actually the gene-rich regions in wheat (Dilbirligi et al., 2004; Erayman et al., 2004; Philippe et al., 2013). Therefore, the candidate loci identified in the present study may be located in the selection hot spots of wheat genome, and are directly under selection, but more likely the marked regions of the genome have been subjected to selection during the long-term evolution.

As shown in Table 4, putative functions for the candidate loci are consistent with a role in adaptation, as most of these genes are involved in processes vital for plant growth and survival under stressful conditions. Interestingly, enzyme activity of the wheat FBA (chloroplast fructose-bisphosphate aldolase) is obviously influenced by temperature and light, is related with plant response in all types of abiotic stresses (Lv et al., 2011; Cai et al., 2016), and appears to be under positive selection. This result may be explained by the known fact that the shady niche is under stresses of lower temperature and lower intensity of solar radiation in contrast to the sun niche during the growing season (October–May) of wild emmer wheat (Nevo et al., 1988; Li et al., 1999, 2002). This observation suggests that the outlier SNP loci and the genome regions subjected to adaptive selection are consistent with known patterns of selection. The identified loci are of potential interest for plant breeders as they likely contribute to the existing differences between the two microclimatic niches, separated by only a few meters of distance.

## Conclusion

In the present study, EST-related SNP markers were used to investigate adaptive genetic divergence in wild emmer wheat between shady and sunny micro-niches, separated apart by a short distance of 2–4 m. The effect of solar radiation resulted from two antagonizing abutting microclimatic stresses, sun and shade, could cause significant genetic differentiation of wild emmer wheat. A total of 21 outlier loci under positive selection were identified by using four *F*_*ST*_-outlier testing algorithms. These outlier SNP markers and the residing genomic regions followed the known patterns of selection. Loci subjected to positive selection may be functionally important, and hence may be involved in adaptation evolution. In a word, the present works highlight both evolutionary theory and application importance of radiation-associated genetic divergence in wheat improvement.

## Author contributions

JR and LC carried out DNA extraction and data analysis, and drafted the manuscript. JR, XJ, and VF worked on revising and finalizing the manuscript. MZ assisted the lab work. FY designed the SNP markers. JW performed the SNP genotyping. XY and DS was involved in revising and finalizing the manuscript. EN originally collected the germplasm and revised manuscript. ML was in charge of SNP design and genotyping work and participated in drafting the manuscript. JP was in charge of the entire research project including grants application, experimental design, germplasm acquisition, outlining, and finalizing the manuscript. All authors read and approved the final version of the manuscript. All relevant authors and institutions have approved the submission for publication of this manuscript; and all persons entitled to authorship have been so named and have agreed to the submitted version of the manuscript.

## Funding

This work was supported by the China National Science Foundation (NSFC) Grant No. 31030055, 30870233 (to JP) and 31672482, the Natural Science Foundation of Hubei Province, China Grant No. ZRY1326, and the Taishan Scholars Program of Shandong Province in China.

### Conflict of interest statement

The authors declare that the research was conducted in the absence of any commercial or financial relationships that could be construed as a potential conflict of interest.
